# Prevalence and the affecting factors on depression, anxiety and stress (DASS) among elders in Qazvin City, in the Northwest of Iran

**DOI:** 10.1186/s12877-023-03908-z

**Published:** 2023-03-31

**Authors:** Abouzar Raeisvandi, Mohammad Amerzadeh, Fatemeh Hajiabadi, Zahra Hosseinkhani

**Affiliations:** 1 Social Determinants of Health Research Center, Research Institute for Prevention of Non-Communicable Diseases, Qazvin University of Medical Sciences, Qazvin, Iran; 2 Metabolic Diseases Research Center, Research Institute for Prevention of Non-Communicable Diseases, Qazvin University of Medical Sciences, Qazvin, Iran

**Keywords:** Depression, Anxiety, Stress, DASS, Elderly, Prevalence

## Abstract

**Background:**

Depression, anxiety, and stress are among the most common mental health disorders of the elderly that affect the health of individuals and society. Considering the growing trend of the elderly population in Iran, this study aimed to determine the prevalence of these disorders and to identify the factors affecting them in the elderly.

**Methods:**

We conducted this cross-sectional study using cluster random sampling on 301 elderly people referred to Qazvin health centers. Data was collected using the Depression, Anxiety, Stress Scale 21 (DASS-21) questionnaire and analyzed through univariate and multivariate linear regression tests with the interaction between variables in STATA Version 14 software. A P-value of less than 0.05 was significant.

**Results:**

The prevalence of depression was 45.5%, anxiety 35.5%, and stress 40.2%. Our findings showed that 22.9%, 7.9%, and 14.3% of people had severe and very severe levels of depression, anxiety, and stress. The variables of age, comorbidity, living status and job status had a significant relationship with the DASS score (*p* < 0.05). There was an interaction between the variables of comorbidity and income status influencing the DASS score (β = 0.68, 95% CI 0.15, 1.22).

**Conclusion:**

The prevalence of depression, anxiety and stress in the elderly was high, indicating the inappropriate status of their mental health. Therefore, it is necessary to take operational steps to reduce some problems in the elderly, prioritize the elderly suffering from concurrent diseases, the unemployed elderly, those who do not have a certain income, and the elderly who live alone.

## Introduction

Aging is becoming a global phenomenon in both developed and developing countries. The elderly population has increased rapidly during the past decades due to two critical factors: the reduction of mortality and fertility rates and improving the quality of life [1, 2].

The World Health Organization (WHO) has predicted that the world’s elderly population will surpass 12% to 22% by 2050 [3, 4]. It is expected that the world’s elderly population will increase to 28% by the end of the twenty-first century [5]. In 2050, 80 percent of the elderly will live in low- and middle-income countries, unprepared to face aging and its social and economic effects [6, 7].

Iran is facing aging due to the decrease in the birth rate in the last two decades, medical and health advances, and the increase in life expectancy. The elderly makes up 10% of Iran’s population and in the next thirty years, this number will reach 30% [6]. The speed of population aging in Iran is very significant, and the country’s population aged 65 and over will triple in 26 years (2023–2049), while this will happen in France, England, and America in 157 years, 100 years and 89 years, respectively [8]. After Bahrain and the United Arab Emirates, Iran ranks third in the world in aging, so in 2030 it will experience an explosion in the elderly population, and 25–30% of the country’s population will be over 50 years old [9, 10].

Mental health is a critical indicator of the elderly’s health status, which is crucial in achieving successful aging and having a good quality of life among the elderly in different societies [11]. While most elderly have good mental health, many are at risk of developing mental disorders, neurological disorders, or substance abuse problems https://www.who.int/news-room/fact-sheets/detail/mental-health-of-older-adults. According to the WHO report, over 20% of the elderly suffer from a mental disorder [12, 13], the most common of which are depression, anxiety, and stress [1, 14]. Depression reduces the quality of life of the elderly and increases their dependence on others. It is estimated that over 350 million people suffer from depression in the world, and it is predicted that severe depression will be one of the most debilitating diseases in the world by 2030 [3].

Anxiety symptoms and disorders are also common among the elderly. They are associated with an increased risk of depression, major medical illnesses, cognitive disorders, poor quality of life, delayed recovery from illnesses, severe disability, and mortality in the elderly [15, 16]. Stress is a significant risk factor for physical and mental health and can affect health directly and indirectly. The studies show that people with a high level of perceived stress are at a high risk of anxiety and depression [17].

In Iran, the prevalence of mental disorders varies from 11.7% to 38.9% in different provinces and groups. The studies indicate increased psychiatric disorders between 1999 and 2015 in Iranian adults [18]. In a study of the prevalence of depression, anxiety, and stress among the elderly in Tehran, one out of every ten elderly people suffers from severe anxiety and depression [11]. Soleimaninejad et al.’s study, conducted on 1133 elderly people in five provinces of Iran, found that the prevalence of depression in Iranian elderly people was 35.7%. In this study, the data extracted from the National Plan for the Health of the Elderly, the prevalence of depression in women (42.3%) was higher than in men (28.5%) [19]. Studies in Iran have shown that the Iranian elderly usually experience moderate anxiety disorders [20].

Considering the growing trend of the elderly population in the country and their mental health problems, planning is necessary. Planning requires knowledge of the current prevalence of these problems and the influencing factors. By understanding the determining factors of these disorders among the elderly, it is possible to design and implement the interventions. Therefore, this study aimed to investigate the prevalence and identify factors affecting depression, anxiety, and stress in the elderly of Qazvin.

## Methods

### Participants

We conducted this cross-sectional study in health centers of Qazvin, Iran, from February to March 2020. The study sample comprised elderly people. Inclusion criteria were: (1) willingness to participate in a research project, (2) being native, (3) having the ability to read and write, (4) being of age 60 or above (5) having no disability with hearing, vision and others. The sample size was estimated 301 elders according to µ1 = 12, µ2 = 12.9, SD1 = 3.8, SD2 = 3.7, α = 0.05 and power of 80% [11].

### Measures

We used two instruments in this study:

### The 21-item depression, anxiety, and stress scales

The Depression, Anxiety, and Stress Scale (DASS) (Lovibond and Lovibond, 1995) contains 21 self-report scales to evaluate negative emotional states in depression, anxiety, and stress [21]. This scale measures the intensity of the significant symptoms of depression, anxiety, and stress. These terms are related to the symptoms of negative emotions (depression, anxiety, and stress). The depression subscale includes statements that measure unhappy mood, lack of self-confidence, hopelessness, worthlessness, lack of interest in involvement in affairs, lack of enjoyment of life, and lack of energy and strength. The anxiety subscale has items to assess physiological overarousal, fears, and situational anxiety. The stress subscale includes difficulty achieving relaxation, nervous tension, irritability, and restlessness. This tool was a Likert-type score with four options from no, low, high, and very high levels. A score of 3, 2, 1 and 0 was assigned to very high, high, low and no, respectively. The validity and reliability of the short form of the DASS (DASS-21) for the Iranian population were validated in 2004 by Ali Sahibi et al. on 1070 men and women [22].

### Demographic information

Demographic information includes age, gender, marital status (married, widow or divorced and single), educational status (illiterate, elementary, high school, undergraduate and postgraduate), job status (employed, retired, unemployed, housewife), income status (with an independent income, without income, with the children helps and with the support organization help) and living status** (**only, with mate and children, with mate only, with children only and with others). This data was collected with a researcher-made checklist.

### Data collection

The sample size was estimated at 301 persons. Considering that the healthcare centers cover the city’s entire population with their services, we considered each health center a cluster, and considering the proportion of the population in each center, the number of elderly people was determined. We used random cluster sampling, and three hundred and one elderly participated in the study. The documents available in the health centers with the list of all elderly people were used, and the required number of people was randomly selected from the existing list. The questionnaires were completed by the elderly in health centers.

### Data analysis

Data was analyzed using STATA Version 14 software. Mean and standard deviation were used to describe quantitative data, and number and percentage to describe qualitative data. Linear regression was used to determine the relationship between study variables. We evaluated the relationships between variables in univariate regression tests and used multivariate regression tests to adjust the effect of confounding variables. Variables such as age, education, comorbidity and job, income, living and insurance status were entered into the model as independent variables in univariate regression analysis. Subsequently, for calculating the adjusted effect of each variable, we entered variables with p-value lower than 0.2 in univariate analysis into the multivariate regression analysis [23]. We tested the assumptions of each of them (multivariate normality and no multi-collinearity) before conducting the tests.

## Results

We performed this descriptive-analytical cross-sectional study on 301 elderly aged 60 years and over in Qazvin city, northeast of Iran.

Three hundred one elderly people, with an average age of 69.86 years and a standard deviation of 7.20 years, participated in this study. Most participants were female (54.2%), married (57.8%), and had no university education (69.4%). Besides, 52.8% were employed or retired, and 59.1% had an independent income. In the study of comorbidity, the highest rate was related to diabetes (33.2%), followed by hypertension (28.6%) (Table 1).

**Table 1:** Demographic characteristics of elderly participants in the elders in 2020, Qazvin, Iran

Characteristics	Variable	Number (%)	Characteristics	Variable	Number (%)
Gender	Female	163(54.2)	Marital status	Married	174(57.8)
	Male	138(45.8)		Widow or divorce	125(41.5)
Income status	With independent income	178(59.1)		single	2(0.7)
	Without income	85(28.2)	Educational Status	Illiterate	62(20.60)
	With the children helps	26(8.6)		Elementary	99(32.89)
	With the support organization help	12(4.0)		High school	48(15.95)
Job status	Employed	34(11.3)		Undergraduate	90(29.90)
	Retired	125(41.5)		Post graduate	2(0.66)
	Unemployed	9(3.00)			
	Housewife	130(43.2)			
Comorbidity					
Diabetes		100(33.2)	Hormonal disease		20(6.6)
Hypertension		86(28.6)	Infectious disease		0
CDC		33(11.00)	Epilepsy		5(1.7)
Asthma		24(8.00)	Multiple sclerosis		13(4.3)
Liver disease		28(9.3)	Mental disease		72(23.9)
Cancer		9(3.00)	Others		52(17.3)

The prevalence of depression, anxiety, and stress was 45.5%, 35.5%, and 40.2%, respectively. Further, 22.9%, 7.9%, and 14.3% of people had severe levels of depression, anxiety, and stress, respectively (Table 2).

**Table 2:** Frequency of depression, anxiety and stress in the elderly based on the levels of variables in 2020, Qazvin, Iran

Variables	Normal n (%)	Mild n (%)	Moderate n (%)	Severe n (%)	Very severe n (%)
Stress	180(59.8)	41(13.6)	37(12.3)	24(8.0)	19(6.3)
Anxiety	194(64.5)	41(13.6)	42(14.0)	23(7.6)	1(0.3)
Depression	164(54.5)	28(9.3)	40(13.3)	38(12.6)	31(10.3)

Univariate analysis to investigate the relationship between the variables and DASS scores indicated a significant relationship between age and DASS (*p* = 0.001). A significant relationship was observed between comorbidity (*p* = 0.001), income status (*p* = 0.003), and insurance status (*p* = 0.008) with the DASS score.

We investigated the relationship between education level and DASS score in five levels of post-graduate education, undergraduate, high school, elementary and illiterate. The illiterate group was considered the base. The DASS score had a significant difference only in the group with a undergraduate degree compared to the base group. People with undergraduate degrees had a lower DASS score than the base group (*p* = 0.003).

In the study of the variable effect of living status on the DASS score, living alone was used as the basis. We observed that people living with their mates and children have a lower DASS score (*p* = 0.007), but the elderly living with children only (*p* = 0.036) or living with others (*p* = 0.008) had a higher DASS score. Finally, we studied the relationship between job status and the DASS score in four categories, employed, retired, unemployed, and housewife. We considered the employed category as the basis. The results indicated higher DASS scores in all three studied categories than in the base category. We observed no significant relationship between the variables of gender and marital status with DASS.

The multiple linear regression model was used to investigate the effect of each independent variable on the DASS score. The results of the test showed a significant effect of age (*p* < 0.001), comorbidity (*p* = 0.001), living status (*p* < 0.005), and job status (*p* < 0.005) on the DASS score.

The final model showed that increasing age (adjusted β = 0.03, 95% CI 0.009, 0.04), comorbidity (adjusted β = 0.41, 95% CI 0.17, 0.67), living with children only (adjusted β = 0.48, 95% CI 0.09, 0.86) and with others (adjusted β = 1.97, 95% CI 0.69, 3.25), unemployment (adjusted β = 0.77, 95% CI 0.09, 1.45) and housekeeping (adjusted β = 0.42, 95% CI 0.06, 0.78) was significantly associated with an increase in DASS score.

The existence of an interaction between the variables was investigated, and the results indicated the existence of an interaction between the variables of comorbidity and income status (β = 0.68, 95% CI 0.15, 1.22) (Table 3).

**Table 3:** Univariate and multiple linear regression analysis between measured covariates and DASS scores in the elderly in 2020, Qazvin, Iran

Variables		Crud B	Adjusted B
Age		0.04(0.02, 0.05) *P* = 0.001	0.03(0.009, 0.04) *P* < 0.001
Education	Illiterate	Ref	-
	Elementary	-0.19(-0.51, 0.12) *P* = 0.235	-
	High school	0.26(0.64, 0.11) *P* = 0.171	-
	Undergraduate	-0.49(-0.82, -0.17) P = 0.003	-
	Post graduate	-1.17(2.56, 0.23) *P* = 0.101	-
Comorbidity	Yes	Ref	Ref
	No	-0.57(-0.81, -0.32) *P* = 0.001	-0.41(-0.67, -0.17) *P* = 0.001
Income status	Independency income	Ref	
	Without income	0.34(0.12, 0.58) *P* = 0.003	-
Living status	Only	Ref	-
	With mate and children	-0.49(-0.84, -0.14) *P* = 0.007	-0.17(-0.51, 0.18) *P* = 0.301
	With mate only	0.004(-0.25, 0.26) *P* = 0.539	0.08(-0.17, 0.32) *P* = 0.531
	With children only	0.43(0.03, 0.83) *P* = 0.036	0.48(0.09, 0.86) *P* = 0.016
	With others	1.83(0.47, 3.19) *P* = 0.008	1.97(0.69, 3.25) *P* = 0.003
Insurance status	No insurance	Ref	-
	With insurance	-0.24(-0.41, -0.06) *P* = 0.008	-
Job status	Employed	Ref	Ref
	Retired	0.45(0.08, 0.82) *P* = 0.017	0.14(-0.22, 0.49) *P* = 0.445
	Unemployed	1.22(0.51, 1.94) *P* = 0.001	0.77(0.09, 1.45) *P* = 0.026
	Housewife	0.72(0.35, 1.09) *P* < 0.001	0.42(0.06, 0.78) *P* = 0.022
Interaction term			
Comorbidity *income	0.68(0.15, 1.22) *P* = 0.013		

## Discussion

Mental disorders are one of the critical issues of aging in the world and Iran, which require essential planning and intervention. Considering the high rate of growth of the elderly population in Iran, the importance of this issue is greater.

In the current study, the prevalence of depression, anxiety, and stress was 45.5, 35.5, and 40.2%, respectively, indicating the high prevalence of these disorders.

In the studies conducted in Iran, a study investigated the prevalence of depression, anxiety, and stress in Yazd adults. The prevalence of these disorders was 29%, 32.2%, and 34.8%, respectively [18]. A study of seven databases reported that the prevalence of depression in the Iranian elderly was about 43% from 2001 to 2015, consistent with our results [24]. In the meta-analysis study by Esfahani et al., conducted in 2019 among the elderly in Iran, the prevalence of depression was 15% based on the random model, with the highest prevalence in Khorramabad city at 82.1% and the lowest in Khoy city with 0.003%. This difference has been attributed to different cultures, environmental factors, hereditary factors, and study methodology [25].

The prevalence of depression, anxiety, and stress in the elderly of Nepal was 15.4%, 18.1%, and 12.1%, respectively, significantly different from our results [26]. The prevalence of these disorders in Ghanaian adults was 25.2%, 53.3%, and 9.7%, respectively. The difference in the results can be attributed to cultural-social factors, economic characteristics, study methods, and different tools [27].

The high prevalence of these disorders in our study indicates the unfavorable mental health status of the elderly. It can be due to the low access of different age groups to mental health services or their lack of information about these centers, which causes a lack of diagnosis of initial symptoms and a lack of detection or late detection of infected people. Due to the fundamental changes in Iranian lifestyle, the transformation of extended families into nuclear families, and the migration of the young generation to the cities, the support structures for the elderly have been weakened, and these people are at a greater risk of suffering from mental disorders.

Considering the coincidence of our study with the Covid-19 pandemic, a part of the prevalence of the disorder in our subjects can be attributed to this pandemic. In Sarbouzi et al.’s study, conducted to investigate depression, anxiety, and stress in the families of nurses during the covid-19 pandemic, the prevalence of depression, anxiety, and stress was 43.7%, 35.6%, and 43.7%, respectively, consistent with our results [28].

In the study of the factors affecting depression, anxiety, and stress in the elderly, age, comorbidity, living status, and income status were significant predictors of the risk of these disorders. Increasing age, even weakly, was associated with increasing DASS scores. With increasing age, it can be expected that most elderly will face problems such as loneliness, financial needs, and comorbidities. As a result, they will be exposed to a greater risk of suffering from mental disorders. Nevertheless, the results of different studies are not the same [29, 30].

The results showed that comorbidity was significantly associated with an increase in depression, anxiety, and stress. This finding is consistent with other studies that reported comorbidity as the main predictor of mental disorders. According to the World Health Organization’s (WHO) Global Burden of Disease Study, people with chronic diseases were significantly more likely to suffer from depression, and that the comorbidity between chronic diseases and depression is common [31]. Elderly people with comorbidity need more support and medical services, expose to more healthcare costs, and are often at a higher risk of being hospitalized [32]. These findings highlight the importance of paying more attention to the impact of chronic diseases on patients’ mental health [31].

In this study, living status were among the factors affecting depression, anxiety, and stress. The results of the univariate analysis showed that the elderly who lived with both their mates and children had higher mental health, and depression, anxiety, and stress were less in them. However, this finding was not significant in multivariate regression. The family plays a central role in supporting its members, and living with a spouse and children is one of the most critical factors in the perception of social support, which can increase life expectancy and plays an influential role in the mental health of the elderly [33].

In this study, living only with children and living with others was associated with an increase in depression, anxiety, and stress. The increase in depression, anxiety, and stress in the elderly who live only with their children or with others can be due to the elderly being deprived of spending time as they wish, taking care of the house, doing housework, insufficient financial and emotional support, lack of attention to their needs, and even domestic violence [1].

We observed no significant difference in the DASS score between working and retired people. It could be because retired people have income (although low) that they are not financially dependent on others or have low dependence. The results showed that unemployment and housekeeping are the factors that increase mental disorders in the elderly. Due to the lack of income, the unemployed elderly need the financial support of other family members or supporting organizations. These people face severe challenges because the elderly is associated with chronic and expensive diseases which can cause or aggravate mental disorders. In the study of Moghadam et al., conducted to investigate the relationship between social support and depression in the elderly, the results indicated a higher rate of depression in the elderly with low income [34].

In this study, gender had no significant effect on DASS. Gender is a socio-demographic factor that is often associated with depression and anxiety. The female gender is significantly associated with the increase in these disorders [35–37]. The factors involved in this difference in old age are not well known [38], and the results of different studies are not the same. For example, Hosseini et al. studied the relationship between depression, anxiety, and stress with perceived social support in the elderly in Iran.The levels of depression and stress in men were higher than in women [14]. In addition, in several studies, no relationship between gender and depression in elderly people was observed [39].

The study’s results indicated a mutual effect between comorbidity and income status in depression, anxiety, and stress. This finding indicates that comorbidity and a lower income have a higher DASS score. As mentioned, aging is associated with the possibility of chronic and expensive diseases. The elderly with comorbidities and who do not have a sufficient income face more problems than others. This issue can have a double effect on increasing their DASS score.

One limitation of this study can be the self-reporting of the elderly, who may not be careful enough to provide the answers. Another point is the cross-sectional nature of this study, meaning that longer studies may yield different or more accurate results. Also due to the coincidence of our study with the Covid-19 pandemic, a part of the prevalence of these disorders in our subjects could be due to the pandemic.

## Conclusion

The high prevalence of depression, anxiety, and stress in the elderly indicates their unfavorable mental health status. Therefore, it is necessary to take operational steps to reduce some problems of the elderly, with priority given to the elderly who are suffering from concurrent diseases, the unemployed and without a certain income, and the elderly who live alone or without a family. We suggest establishing laws per the country’s conditions and providing the basis for the active participation of the elderly in various programs and activities to use their experiences. To strengthen the capacity of donors and non-governmental organizations, activities and programs related to mental health, the information on these services at the community level should be expanded to prevent these disorders, and timely identification of affected people and their effective treatment is essential. Educational programs should be conducted for self-esteem and to strengthen the abilities of the elderly.

## Data Availability

The dataset of the current study is available from the corresponding author (Zahra Hosseinkhani).
